# Unhealthy fantasizing of a loved person who does not exist in reality

**DOI:** 10.1192/j.eurpsy.2022.1657

**Published:** 2022-09-01

**Authors:** S. Nahar

**Affiliations:** National Institute of Mental Health, Dhaka, Bangladesh., Adult Psychiatry, Dhaka, Bangladesh

**Keywords:** Self talking, Unhealthy fantasizing

## Abstract

**Introduction:**

Obsessive-compulsive disorder is a disorder in which recurring, intrusive, unwanted thoughts, ideas or sensations cause them to feel driven to do something repetitively. The repetitive behaviors can significantly interfere with a person’s daily activities and social interactions. Not performing the behaviors commonly causes great distress.

**Objectives:**

Clinical trials are required for proper assessment and management of this group of patients.

**Methods:**

Mrs. X, a 28 year old female coming for psychiatric consultation with the complaints of self talking, irritability and aggression, insomnia.She fantasizes a man publicly, whom she loves but he has no existence in reality. She tries to solve all kinds of problems by talking to that fantasy man. If anyone interrupts her, she becomes irritable, shouts and breaks things. But for the last 3 months she realized that its a problem. Because, she can’t eat, sleep and concentrate on her household chores for this fantasy. Even she can’t take care of her child and feeling low sexual affection for her husband. On personal history, she said that she had poor attachment with her parents.

**Results:**

After thorough assessment, her consultant Psychiatrist told that she was sufering from unhealthy fantasy obsession or Obsessive Compulsive Disorder.

**Conclusions:**

It is very important and urgent to assess and manage this group of patients, because this has a devastating impact on relationships, specially on the conjugal life. Psychoeducation about the ilness and Psychotherapy along with pharmacotherapy should be the mode of treatment.

**Disclosure:**

No significant relationships.

